# 

**DOI:** 10.1192/bjb.2024.121

**Published:** 2025-12

**Authors:** Nigel Wilkes

**Affiliations:** ADIRA Wellness Centre, Sheffield, UK. Email: nwilkes48@gmail.com

**Figure:**
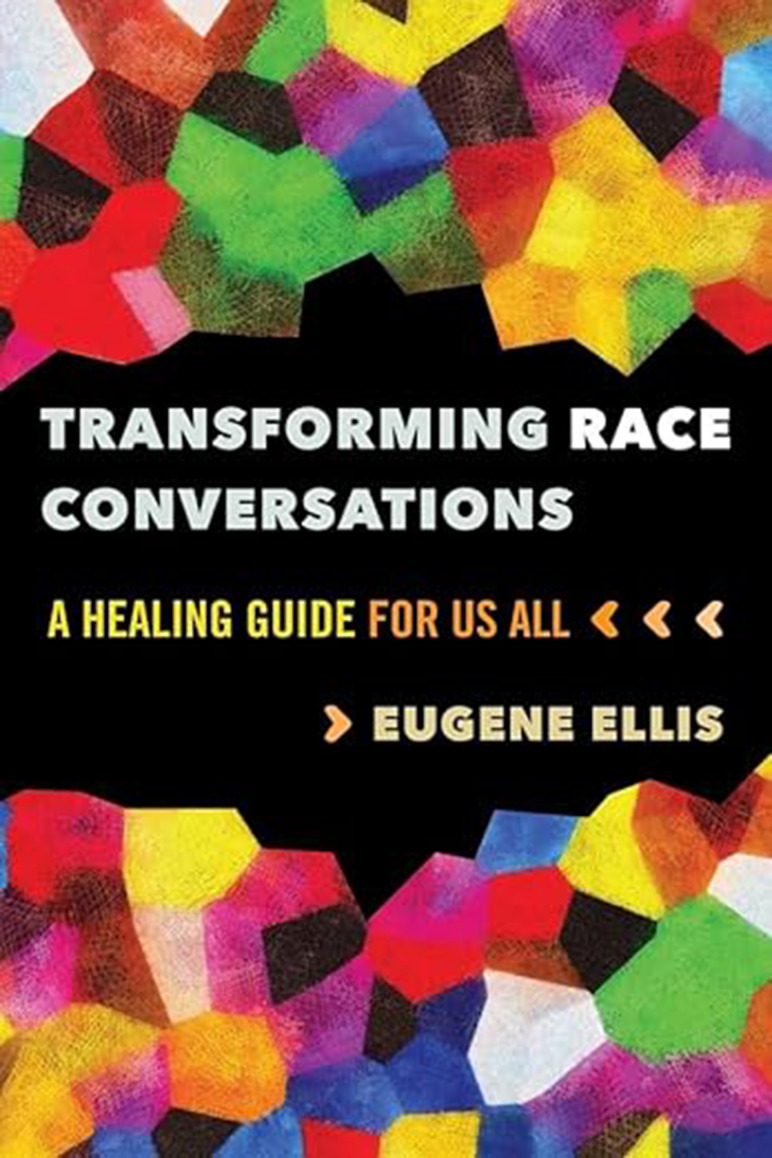

*Transforming Race Conversations* by Eugene Ellis is an exploration of the complexities of race in modern-day society. Ellis is a psychotherapist with a lot of experience of working with diverse communities. He offers a unique perspective that blends personal reflection and historical content, and gives us some practical strategies that can be used to enable us to navigate difficult conversations.

The book is divided into eight chapters, and each chapter title is supplemented by subheadings that give more insight into the subject matter of the chapter. The language used throughout is accessible not just to academics, but also to those who have an interest in exploring the barriers associated with this topic, and are willing to be open and honest about the fears we all have when tackling this subject.

The book takes us on a historical journey that goes into the emotional and psychological impacts of racism. Ellis challenges the reader to confront their internalised beliefs and assumptions, encouraging them to develop a deeper understanding of their own privileges or disadvantages.

These conversations will awaken the emotions within all of us due to the generational loading that has been passed down over the years and is deeply embedded within those from the African–Caribbean culture.

As African–Caribbeans, we are already primed for the arousal: macro- and microaggressions that put us into a state of fear and the feeling that we need to defend ourselves when conversations about race are brought up. The feelings of superiority felt by White people is sensitively explained from a historical context through, among other things, the connection to slavery.

Ellis goes on to explain race as a social construct, and how and why it was created. This allows the reader to gain an understanding of why these conversations are difficult, and that by understanding some of the background information we may begin to have an open and honest dialogue.

Although the book does provide valuable insight into how we can engage in more productive conversations about race, there is still the challenge of how we undo some of the narratives that people have acquired as a result of years of conditioning. Approaching the conversation with a willingness to listen, and being prepared, in some instances, to challenge previous narratives is needed to take advantage of the information presented in this book.

Overall, *Transforming Race Conversations* is a valuable resource for those who are committed to fostering greater understanding and empathy across racial divides. It offers a nuanced and compassionate approach to a complex and often divisive topic.

